# Supplementary material for: Spontaneous eye blink rate indicates increased attention during grooming in female Barbary macaques
Source: Sci Rep. 2026 May 28;16:16556. doi: 10.1038/s41598-026-53538-9 (PMC13219460; doi:10.1038/s41598-026-53538-9)
Supplement: Supplementary file 1 — Supplementary Information. [file 41598_2026_53538_MOESM1_ESM.docx]

Supplementary Information

Table 1: Description of behaviors recorded during instantaneous group scans

| **Activity** | **Partner** | **Definition** |
| --- | --- | --- |
| active grooming | ID of grooming partner recorded | Subject goes through the fur of another individual with its fingers, occasionally removing dirt or parasites. |
| being groomed | ID of grooming partner recorded | Subject is being groomed by another individual. |
| resting | If in body contact, partner ID is recorded. | Subject rests by sitting or lying down and is not  engaged in any other activity (feeding, travelling, grooming). Resting can also occur in body contact. |
| feeding |  | Subject forages by stroking the ground,  Touches searching for food, lifting or ingesting food items. |
| traveling |  | Subject moves forward with a walking gait  or faster for over 5s. |
| 1.5 m proximity | ID of partner in 1.5m recorded. | Another individual is within 1.5m distance to subject (irrespective of subject’s activity) |

Table 2: Description of behaviors recorded during ad libitum sampling for the quantification of dominance ranks

| **Behavior** | **Type** | **Definition** |
| --- | --- | --- |
| make room | submission | Subject makes (the beginning) of a movement away from  another individual. Often only the upper body is moved, the monkey never moves over a large distance. May occur without preceding aggression by another individual (unprovoked). |
| give ground | submission | Subject creates a distance between itself and another individual by moving away, yet not at full speed. May occur without preceding aggression by another individual (unprovoked). |
| flee | submission | Subject runs away from another individual at full speed; always preceded by aggressive behavior by another individual. |
| crouch | submission | Subject presses itself to the ground, tucking its arms, legs and head under its body. |
| open mouth | aggression | Body is tense, usually head is lowered and stuck forward. Eyes are wide open; subject stares directly at another individual. Ears  are held out away from head, sometimes eyebrows are lifted. Mouth is opened, jaws are tense, lips cover the teeth. |
| stare | aggression | Body is tense, usually head is lowered and stuck forward. Eyes are wide open; subject stares directly at another individual. Ears  are held out away from head, sometimes eyebrows are lifted. |
| lunge | aggression | Subject makes a sudden, intense movement towards another individual. It does not move over a large distance, sometimes only the upper body is moved. |
| head bop | aggression | Subject moves its head up and down in short, intense movements. |
| chase | aggression | Subject runs after another fleeing individual at high speed. |
| point | aggression | Subject stretches its body while staring directly at another individual. Head is in line  with rest of the body, eyebrows are  lifted, ears are held flat against the  head. Eyes are half-closed, so that the  white “flags" on the eyelids show. Mouth  forms a triangle. |
| slap | aggression | Subject hits another individual with an open hand. |
| ground slap | aggression | Subject slaps the ground with an open hand in short, intense movements. |
| bite | aggression | Subject uses its teeth to grab another individual in skin and fur. |
| open mouth with bared teeth (silent scream face) | aggression | Subject’s body is tense; usually head  is lowered and stuck forward. Eyes are  wide open staring directly at another individual. Ears are held out away from head, sometimes eyebrows are lifted. Mouth  is opened, jaws are tense and lips are  pulled up, so that teeth and sometimes gums are shown. |

Table 3: Effect of activity type on eye blink rate (GLMM, subset of resting events not terminated due to eye closure of the subject; N grooming = 229, N resting = 135)

| Term | Estimate | SE | 95% CI | ChiSq | Df | p-value |
| --- | --- | --- | --- | --- | --- | --- |
| Intercept | -1.767 | 0.047 | [-1.859, -1.675] |  |  |  |
| Activity type (resting)^1^ | 0.523 | 0.040 | [0.445, 0.601] | 37.090 | 1 | <0.001 |
| Subject age^2^ | 0.027 | 0.049 | [-0.068, 0.123] | 0.313 | 1 | 0.576 |
| Subject rank^3^ | -0.074 | 0.045 | [-0.161, 0.014] | 2.454 | 1 | 0.117 |

*Note*: Indicated are model estimates, standard errors, 95% confidence limits, likelihood ratio test, and significance test.

^1^Activity type was dummy coded with grooming being the reference level

^2^Subject age and rank were z-transformed to mean = 0 and SD = 1.

Modified model 2 (categorical rank difference between groomer and groomee)

We ran a slightly modified model 2 with a categorical predictor of rank difference (rank direction: up/down the hierarchy) instead of a continuous measure of rank difference. Again, the full-null model comparison did not reveal significance (χ^2^ = 0.418, df = 2, P = 0.811). Correspondingly, neither dyadic affiliative relationship strength nor dyadic dominance rank direction were significant predictors of eye blink rate during active allo-grooming (Table 4).

Table 4: Effect of dominance rank direction (up or down the hierarchy) and dyadic affiliative relationship strength (DSI) on eye blink rate during grooming interactions (GLMM)

| Term | Estimate | SE | 95% CI | ChiSq | Df | p-value |
| --- | --- | --- | --- | --- | --- | --- |
| Intercept | -1.757 | 0.054 | [-1.861, -1.652] |  |  |  |
| Rank direction ^1^ | -0.021 | 0.038 | [-0.095, 0.053] | 0.297 | 1 | 0.584 |
| Relationship strength^2^ | -0.003 | 0.038 | [-0.077, 0.072] | 0.006 | 1 | 0.939 |
| Subject age^3^ | -0.011 | 0.056 | [-0.121, 0.098] | 0.040 | 1 | 0.841 |

*Note*: Indicated are model estimates, standard errors, 95% confidence limits, likelihood ratio tests, and significance tests.

^1^Categorical rank direction (subject – groomee)

^2^Relationship strength was z-transformed to mean = 0 and SD = 1; mean and SD of original affiliative relationship strength were 3.53 and 3.57.

^3^Subject age was z-transformed to mean = 0 and SD = 1; mean and SD of original rank were 14.92 and 5.63, respectively
